# Implementation of a national smoke-free prison policy: an economic evaluation within the Tobacco in Prisons (TIPs) study

**DOI:** 10.1136/tobaccocontrol-2021-056991

**Published:** 2022-03-07

**Authors:** Nicola McMeekin, Olivia Wu, Kathleen Anne Boyd, Ashley Brown, Emily J Tweed, Catherine Best, Peter Craig, Alastair H Leyland, Evangelia Demou, Tom Byrne, Jill Pell, Sean Semple, Helen Sweeting, Lesley Graham, Kate Hunt

**Affiliations:** 1 HEHTA, Institute of Health and Wellbeing, University of Glasgow College of Medical, Veterinary and Life Sciences, Glasgow, UK; 2 Institute for Social Marketing and Health, University of Stirling, Stirling, UK; 3 MRC/CSO Social and Public Health Sciences Unit, University of Glasgow, Glasgow, UK; 4 Healthcare Improvement Scotland, Glasgow, UK; 5 Institute of Health and Wellbeing, University of Glasgow, Glasgow, UK; 6 Public Health Scotland, Edinburgh, UK

**Keywords:** economics, public policy, secondhand smoke

## Abstract

**Objective:**

To determine the cost-effectiveness of a smoke-free prison policy in Scotland, through assessments of the trade-offs between costs (healthcare and non-healthcare-related expenditure) and outcomes (health and non-health-related non-monetary consequences) of implementing the policy.

**Design:**

A health economic evaluation consisting of three analyses (cost-consequence, cost-effectiveness and cost-utility), from the perspectives of the healthcare payer, prison service, people in custody and operational staff, assessed the trade-offs between costs and outcomes. Costs associated with the implementation of the policy, healthcare resource use and personal spend on nicotine products were considered, alongside health and non-health outcomes. The cost-effectiveness of the policy was evaluated over 12-month and lifetime horizons (short term and long term).

**Setting:**

Scotland’s national prison estate.

**Participants:**

People in custody and operational prison staff.

**Intervention:**

Implementation of a comprehensive (indoor and outdoor) smoke-free policy.

**Main outcome measures:**

Concentration of secondhand smoke, health-related quality of life (health utilities and quality-adjusted life-years (QALY)) and various non-health outcomes (eg, incidents of assaults and fires).

**Results:**

The short-term analyses suggest cost savings for people in custody and staff, improvements in concentration of secondhand smoke, with no consistent direction of change across other outcomes. The long-term analysis demonstrated that implementing smoke-free policy was cost-effective over a lifetime for people in custody and staff, with approximate cost savings of £28 000 and £450, respectively, and improvement in health-related quality of life of 0.971 QALYs and 0.262, respectively.

**Conclusion:**

Implementing a smoke-free prison policy is cost-effective over the short term and long term for people in custody and staff.

## Introduction

People in custody (PiC) disproportionately come from deprived communities, where smoking rates are around four times higher than the most affluent areas.^1 2^ Globally, smoking prevalence in prisons is around two to eight times higher than in the general population.^3^ A 2017 survey in Scotland showed that smoking rates among PiC in Scottish prisons were reported to be 68%,^4^ almost four times higher than that of the Scottish general population.^5^ This high smoking prevalence contributes to poor health, directly among PiC who smoke, and indirectly among PiC and prison staff through exposure to secondhand smoke (SHS).^6^

Legislation on smoke-free public places substantially reduces SHS exposures^7 8^ and improves health outcomes for smokers and those previously exposed to SHS.^6 9^ Expanding smoke-free policies to prison settings may similarly improve the health of prison staff and PiC and has been shown to reduce numbers of smoking relating deaths for PiC.^7^ This improvement in health may reduce health inequalities for PiC and likely reduce expenditure for health services. There is also potential for PiC to benefit financially if they are no longer purchasing tobacco. However, these policies could conversely result in additional costs, such as provision of smoking cessation support and responding to adverse outcomes should serious unrest occur.

Prior to the introduction of the smoke-free policy (hereafter referred to as ‘the policy’), PiC were able to purchase tobacco products from the prison shop (canteen), including rolling and pipe tobacco, cigarettes, lighters, filters, papers and rolling machines. Electronic cigarettes (e-cigarettes), chargers and e-liquids became available for purchase from canteen lists 2 months prior to the implementation of the policy and have remained available since then. In addition, e-cigarette starter packs were available free of charge, for a limited period, to eligible smokers entering custody. Where possible, PiC who do not use e-cigarettes are not allocated to share a cell with an e-cigarette user. Prison staff and visitors to prisons in Scotland have not been permitted to smoke (or vape) on prison premises for many years.

While limited evidence on the health benefits of smoke-free prison policies exists,^7 10^ there is none relating to the cost-effectiveness of these policies.^3 11^ This paper reports an economic evaluation of introducing a smoke-free prison policy in Scotland, conducted as part of the Tobacco in Prisons (TIPs) study, which was the first multimethod, multiphase evaluation of its kind across a national prison system. TIPs findings on SHS levels in prisons partially informed the Scottish Prison Service’s (SPS) decision to implement the smoke-free policy.^12^

The aim of this health economic evaluation was to determine the cost-effectiveness of a smoke-free prison policy, compared with the absence of such a policy, through assessments of the trade-offs between costs (healthcare and non-healthcare expenditure) and outcomes (non-monetary health and non-health consequences).

## Methods

In health economic evaluations, a cost-utility analysis (using a health-related quality of life outcome) is typically conducted to assess the trade-off between costs and outcome of an intervention and inform decision makers. However, in public health interventions, impacts of the intervention often go beyond health. In line with the National Institute for Health and Care Excellence (NICE) recommendations on broadening approaches to evaluating public health interventions,^13^ the health economic evaluation consisted of three complementary analyses (table 1): (1) cost-consequence analysis to capture broader impacts of the policy beyond a single health outcome measure, (2) cost-effectiveness analysis to determine the value of the policy on the reduction of SHS, a key outcome in the TIPs study, and (3) cost-utility analysis to determine the value of the policy on health-related quality of life and benchmark against NICE’s current cost-effectiveness threshold of £20 000 per quality-adjusted life-year (QALY) gained.

**Table 1:** Summary of evaluation methods

Type of economic evaluation	Costs/outcomes	Justification for inclusion	Sources	Cost-effectiveness measure
Cost-consequence analysis comparing three phases (preannouncement vs preparatory vs post implementation)	**Costs (healthcare and personal spend on nicotine products)**			No cost-effectiveness measure presented—balance sheet format presenting disaggregated costs and outcomes
	GP/nurse visits (PiC and staff)	Potential for change with reduced exposure to tobacco/SHS (eg, for coughs and colds)	TIPs surveys of PiC and staff (all three phases)	
	Outpatient visits (PiC only)	Potential for change with reduced exposure to tobacco/SHS for smoking-related diseases	NHS NSS (ISD SMR00) (June 2016 to November 2019)	
	Inpatient stays (PiC only)	Potential for change with reduced exposure to tobacco/SHS for smoking-related diseases (eg, stays for cardiovascular events and respiratory disease)	NHS NSS (ISD SMR01) (June 2016 to November 2019)	
	Mental health stays (PiC only)	Potential for change with no licit access to tobacco, which could impact levels of distress	NHS NSS (ISD SMR04) (June 2016 to November 2019)	
	Accident and emergency (PiC only)	Potential for change with reduced exposure to tobacco/SHS for smoking-related diseases (eg, acute health events) and with no licit access to tobacco (eg, violence, including self-harm)	NHS NSS (ISD Unscheduled Care A&E2) (June 2016 to November 2019)	
	Ambulance (PiC only)	Potential for change with reduced exposure to tobacco/SHS for smoking-related diseases (eg, acute health events) and with no licit access to tobacco (eg, violence, including self-harm)	Scottish Prison Service (June 2016 to November 2019)	
	Medication—nicotine dependence and smoking-related illness (PiC only)	Potential for change with reduced exposure to tobacco/SHS (need for medication for smoking-related diseases), and with no licit access to tobacco (need for nicotine dependence products)	National Procurement, NHS NSS (June 2016 to November 2019)	
	Tobacco products (PiC and staff)	PiC—expected decrease when unavailable in canteen after implementation; staff—potential change in spend if influenced by policy	PiC—SPS canteen purchase data (3 months prior to implementation); staff—TIPs staff survey all three phases	
	E-cigarettes (PiC only)	Expected increased use with no licit access to tobacco in later phases	SPS canteen purchase data (3 months prior to implementation and 1 year after)	
	**Outcomes (health and non-health related)**			
	Concentration of secondhand smoke (PM_2.5_)	Expected reduction due to policy implementation	TIPs study measurements (in all three phases)	
	Health-related quality of life—health utilities (PiC and staff)	Potential for change for PiC and staff due to reduced exposure to SHS and no licit access to tobacco	TIPs surveys for PiC and staff included a Euro-Qol-5D (EQ-5D) questionnaire (in all three phases)	
	Prisoner-on-staff assaults	Potential for change with no licit access to tobacco later in preparatory and post implementation phases	Scottish Prison Service (November 2017 to November 2019)	
	Prisoner-on-prisoner assaults	Potential for change with no licit access to tobacco later in preparatory and post implementation phases	Scottish Prison Service (November 2017 to November 2019)	
	All-cause mortality (deaths in custody—PiC)	Potential for change with reduced exposure to tobacco/SHS for smoking-related diseases	Scottish Prison Service (June 2016 to November 2019)	
	Fires	Potential for change due to no lighters permitted after implementation and frustration at no licit access to tobacco	Scottish Prison Service (June 2016 to November 2019)	
	Management of an Offender at Risk due to any Substance (MORS) policy	Potential for change with no licit access to tobacco and with the introduction of e-cigarettes in prisons	Scottish Prison Service (June 2016 to November 2019)	
Cost-effectiveness analysis comparing absence and presence of smoke-free policy (preannouncement to post implementation phases)	**Costs**—total of all costs included in cost-consequence analysis	Potential for change due to absence of licit tobacco—details above	Various sources—details above	Incremental cost per 10 µg/m^3^ reduction in PM_2.5_
	**Outcome**—concentration of secondhand smoke (PM_2.5_)	Expected reduction due to policy implementation	TIPs study measurements (in all three phases)	
Cost-utility analysis comparing absence and presence of smoke-free policy (preannouncement to post implementation phases)	**Costs**—total of all costs included in cost-consequence analysis	Potential for change due to absence of licit tobacco—details above		Incremental cost per quality-adjusted life-year
	**Outcome**—quality-adjusted life-years (PiC and staff)	Potential for change for PiC and staff due to reduced exposure to SHS and no licit access to tobacco	TIPs surveys for PiC and staff included an EQ-5D questionnaire (in all three phases) combined with 12-month time period	

A&E, accident and emergency; GP, general practitioner; ISD SMR, Information Services Division Scottish Morbidity Records; NHS NSS, National Health Service National Services Scotland; PiC, people in custody; PM, particulate matter; SHS, secondhand smoke; SPS, Scottish Prison Service; TIPs, Tobacco in Prisons.

### Cost-consequence analysis

**Figure 1:**
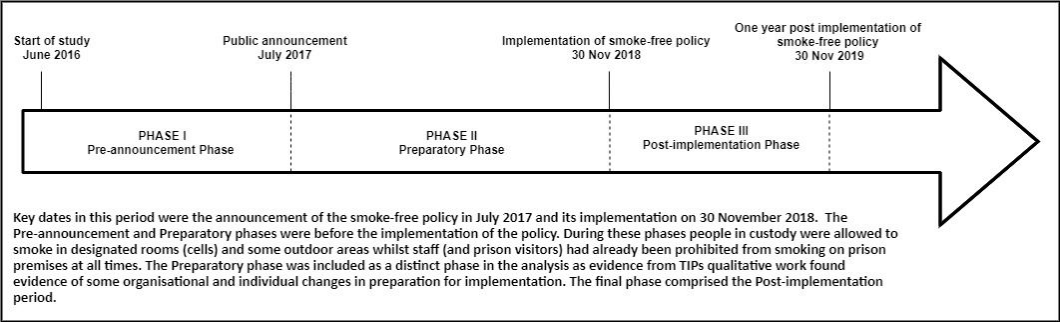
Tobacco in Prisons (TIPs) timeline.

The cost-consequence analysis, based on the perspectives of the National Health Service (NHS), SPS, PiC and prison staff, was used to assess the impact of the policy on a broad range of relevant outcomes (beyond the usual QALY outcome). The changes in costs and relevant outcomes, between the three phases of TIPs (preannouncement, preparatory and post implementation; figure 1) were evaluated to capture transitional impacts across the entire study period, and are presented in a balance sheet format.^14^

#### Tobacco in Prisons

TIPs was a natural experiment based on a wide range of bespoke and routine data collected pre-implementation and post implementation of the smoke-free policy in November 2018. Findings on SHS exposures, dispensed medication and the views and experiences of PiC and prison staff, including on the introduction, use and sale of e-cigarettes in the lead up to implementation, are reported in detail elsewhere.^12 15–26^ Data collected in the TIPs study were used to inform this analysis. The populations included were PiC and operational prison staff in Scottish prisons. Operational staff (hereafter referred to as ‘staff’) are based in areas of the prisons (most notably residential halls) where, prior to the policy, they would be exposed to SHS during working hours. Non-operational staff were excluded as they are unlikely to have been regularly exposed to SHS at work.

The Scottish prison estate comprises 14 closed and one open prison. Where the data allowed, only PiC in closed prisons were included in the analysis because PiC in Scotland’s open prison can spend time in the wider community (eg, for work or for home visits) where they have access to tobacco. However, staff at the open prison were included in the analysis as they are affected by the policy during the working day. Information on the range of costs and outcomes that were available separately for the open and closed prisons is included in online supplemental appendix 1.

10.1136/tobaccocontrol-2021-056991.supp1 Supplementary data

#### Resource measurement and valuation

Resource use categories were healthcare and personal nicotine use. We were unable to collect data on implementing the policy (intervention costs). Healthcare resource use associated with general practitioner (GP) and nurse visits was derived from TIPs surveys completed by PiC and staff, which provided mean estimates for each of the three phases of the study period.^17 18 26^ For PiC, routinely collected data (monthly) from SPS and NHS National Services Scotland (NSS) were used to estimate resource use associated with secondary healthcare, including new outpatient visits (receiving treatment in hospital but not requiring an overnight stay), inpatient stays (receiving treatment in hospital requiring an overnight stay), mental health hospital stays (receiving treatment requiring an overnight stay at a mental healthcare unit), accident and emergency visits, ambulance use and dispensed medication for nicotine dependence and smoking-related illness; detailed analysis is described elsewhere.^19^

Unit costs were applied to all healthcare resource use to calculate direct medical costs, except for the cost of PiC medications which were included in the relevant NHS NSS routine data. All costs are reported in 2017/2018 pound sterling (GBP).

Detailed information on resource use data identification, sources, formats and unit costs is reported in online supplemental appendix 2.

PiC personal spend, at a monthly level, associated with tobacco use and e-cigarette products was derived from a complementary Cancer Research United Kingdom (CRUK)-funded analysis of prison canteen data. Mean personal spend associated with staff tobacco use for each of the three phases of the study was derived from TIPs surveys.

#### Outcome measurement and valuation

All outcomes expected to be impacted by the policy implementation were determined a priori in consultation with the broader TIPs research team. These included SHS levels, prisoner and staff health-related quality of life and non-health outcomes.

SHS levels were measured as part of the TIPs study at three time points using fixed-site monitoring of fine particulate matter (PM_2.5_) concentrations inside prisons; details are published elsewhere.^12 15 16^ PM_2.5_ is an air pollutant widely accepted as a proxy for indoor SHS levels.^16^

Health-related quality of life was measured using the five-level Euro-Qol-5D (EQ-5D-5L) questionnaires included in the TIPs surveys at three time points. The EQ-5D-5L measures health-related quality of life through five domains: mobility, self-care, usual activities, pain/discomfort and anxiety/depression. Responses were mapped to the EQ-5D-3L using a mapping algorithm,^27^ as recommended by NICE,^28^ to estimate individual health utilities, which are bounded between ‘0’ (representing death) and ‘1’ (representing full health).

Non-health outcomes include incidents of assaults (prisoner on staff and prisoner on prisoner), number of deaths in custody, incidents of fires and number of PiC included in the Management of an Offender at Risk due to any Substance (MORS) policy. (The MORS policy supports staff to provide appropriate management and care to those suspected of being at risk due to any substance.) These outcomes were recorded at monthly intervals. Further information on outcomes, their sources and formats is available in online supplemental appendix 3.

#### Analysis

Mean costs and outcomes per person per month were estimated for each of the three TIPs study phases and presented separately in a balance sheet format. As previously described, data comprised two formats: monthly, and point estimates for each phase. Interrupted time series analyses were conducted for monthly data (all costs associated with healthcare resource use (except GP/nurse visits), e-cigarette use and non-SHS non-health outcomes).^29^ We included lags where autocorrelation was present and controlled for overcrowding, measured as a monthly ratio of prison population to available contracted places. Controlling for overcrowding was important as the prison population increased over the study period while the contracted available places remained mainly constant and this may have accounted for some changes in costs and outcomes. For point estimate data (GP/nurse visits, personal spend on tobacco, SHS levels and health utilities), a regression framework analysis was conducted. Ordinary least squares regression was used to estimate changes between phases. To explore uncertainty, in addition to the already described base case analysis (using preferred data and most likely assumptions), we also conducted two sensitivity analyses: the first included all dispensed medication (not restricted to nicotine dependence and smoking-related illness); the second included all PiC data from the open prison. Results are presented as an observed monthly mean for costs and outcomes and the measure of change between phases, plus 95% CI for this change.

### Cost-effectiveness and cost-utility analyses

In addition to the cost-consequence analysis, cost-effectiveness (CEA) and cost-utility (CUA) analyses were also conducted from the perspective of the NHS, PiC and staff. In these analyses, mean costs and outcomes (estimated in the cost-consequence analysis) in the preannouncement phase were compared with post implementation phase (using 12-month periods), and incremental cost-effectiveness ratios were estimated, where relevant. Two sensitivity analyses were also conducted, as described in the cost-consequence analysis.

In the cost-effectiveness analysis, cost-effectiveness was expressed as incremental cost per 10 µg/m^3^ reduction in PM_2.5_. This increment in PM_2.5_ was chosen as it is applied by the WHO when assessing mortality risk and has been used in a previous economic evaluation.^30^

Similarly, in the cost-utility analysis, cost-effectiveness was expressed as incremental cost per QALY gained. The mean health utilities estimated in the cost-consequence analysis were combined with the 12-month timeframe of the analysis to generate QALYs. Further, a scenario analysis was also conducted to estimate the long-term cost-effectiveness of the policy. A cohort model-based analysis was conducted to estimate the cost-effectiveness of the policy over a lifetime (to death).

**Figure 2:**
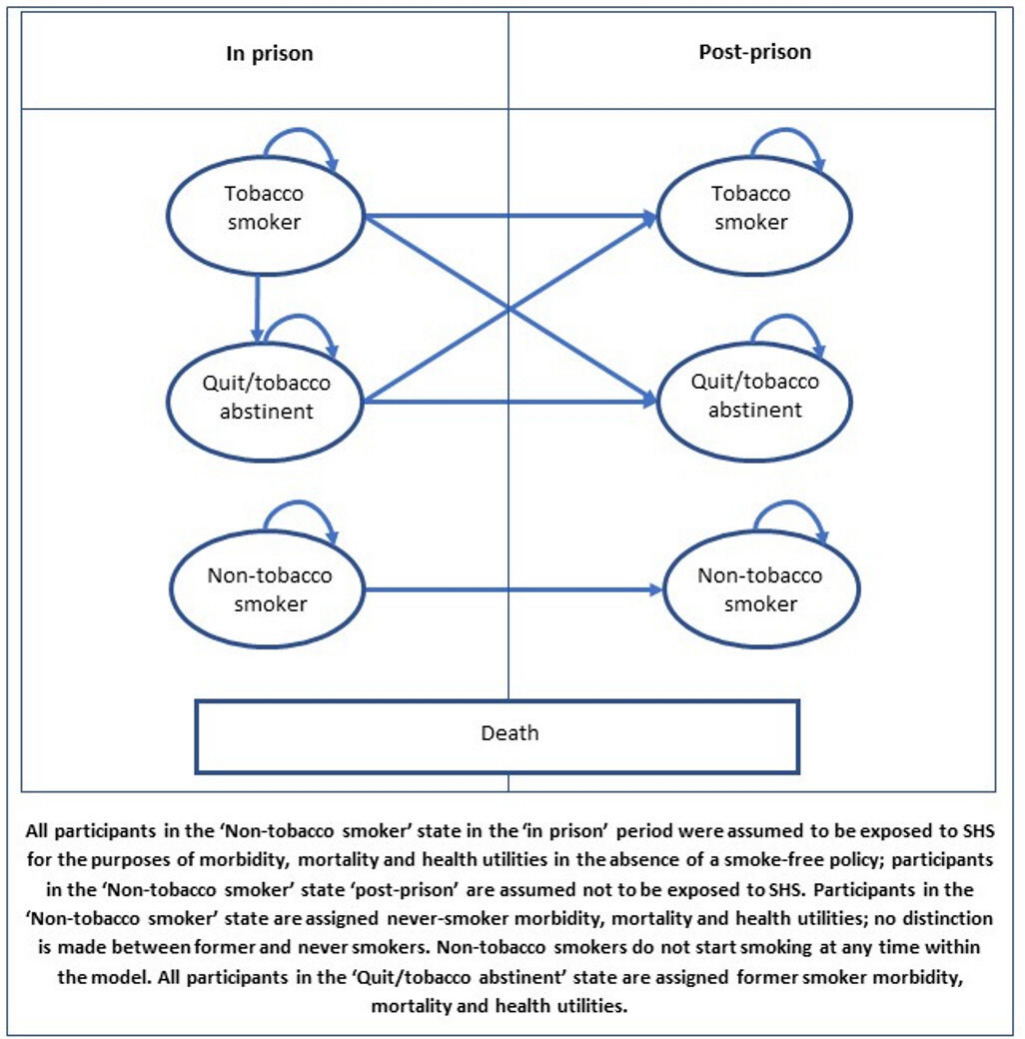
Markov model structure. SHS, secondhand smoke.

A Markov model was developed consisting of seven health states: smoking, quit/abstinent from smoking, no smoking (never smokers) during an ‘in prison’ period, the same states in a ‘post-prison’ period and death (figure 2). PiC enter the ‘post-prison’ period after release, staff after stopping working for SPS.

Two cohorts (PiC and staff) enter the model and accumulate costs and QALYs annually over a lifetime (up to 70 years in the model). Costs and QALYs beyond the first year were discounted at 1.5% following current NICE recommendations for public health interventions.^13^

Full details of the model and model inputs are included in online supplemental appendix 4.

For the base case analysis, where possible, parameters were sourced from TIPs surveys and SPS reports and information. Where there were gaps in the evidence other sources were used, including literature and expert opinion. Key parameters for transitioning between states in the model include smoking status, morbidity and mortality.

To account for parameter uncertainty probabilistic sensitivity analysis was undertaken with 1000 iterations, until model convergence.^31^ Results were plotted on a cost-effectiveness plane to visually represent uncertainty. Several plausible sensitivity analyses were conducted to assess the impact of varying input parameters (parameters reported in online supplemental appendix 4). Per-person base case results were extrapolated to population level for each cohort.

### Patient and public involvement

The overall design of the TIPs study, the development of survey instruments and the sampling for the qualitative data were informed by discussions with prison staff, including those working in SPS headquarters and in the prisons, representatives of prison worker unions and of Scottish Government, and NHS staff, in particular those with a remit for prison health and/or smoking cessation.^26^

## Results

### Cost-consequence analysis

In the base case analysis most mean direct medical costs decreased (outpatient, inpatient, mental health hospital stays, accident and emergency visits, staff GP visits and smoking-related illness medication), but a small number increased (ambulance use, nicotine dependence medication and GP/nurse visits (PiC)). For personal spend, tobacco spend for PiC remained constant across the first two phases (after which tobacco was no longer available for purchase in prison canteens). PiC e-cigarette and staff tobacco spend increased.

For base case outcomes there was a marked decrease in SHS exposure (improvement in air quality), but this was not reflected in PiC health utilities which showed a decrease in self-reported health-related quality of life over all phases. This change in PiC health utilities was largely due to responses to the anxiety and depression domain of the EQ-5D-5L. Staff health utilities increased across all phases showing an improvement in health-related quality of life. For the non-health outcomes, the number of prisoner-on-staff assaults and all-cause mortality (deaths in custody) remained constant, the number of prisoner-on-prisoner assaults and PiC managed under the MORS policy showed an increase and the number of fires showed an increase in the preparatory phase and a decrease in the post implementation phase.

As expected, the impact of the policy was visible in the preparatory phase with changes in costs and outcomes, mirroring qualitative TIPs research suggesting PiC were changing some behaviours before the implementation of the policy. See online supplemental table 1 for phase means and time series figures, and online supplemental appendix 5 for changes between phases. Sensitivity analysis results are also included in online supplemental appendix 5 and showed no major impact on results.

10.1136/tobaccocontrol-2021-056991.supp2 Supplementary data

### Cost-effectiveness and cost-utility analyses

The cost-effectiveness and cost-utility use the same base case costs. These show that PiC total costs were higher overall in the absence of the policy compared with the presence of the policy (£3142 vs £3075) (online supplemental appendix 6). However, some individual cost categories for PiC were higher with the policy: GP/nurse visits, mental health hospital stays, ambulance use, medication for nicotine dependence and e-cigarettes. All staff costs were higher in the absence of the policy compared with the presence of the policy. These results show that implementing the policy is cost saving over the short term for PiC and staff.

The outcome of mean PM_2.5_ (10 µg/m^3^) indicated significantly better air quality with the policy than without it (0.31 compared with 3.84 PM_2.5_ (10 µg/m^3^)), as previously reported.

Therefore, cost-effectiveness results show that, for both PiC and staff, the policy was less costly with a better air quality outcome than the absence of the policy (a dominant strategy), although the difference in costs was small.

In the cost-utility analysis, QALYs decreased for PiC and increased for staff (reflecting the cost-consequence analysis results). The base case incremental cost per QALY for PiC was £1241, and implementing the policy is associated with reduced costs but also reduced QALYs (losing self-reported health benefits). For staff, implementing the policy was dominant (costs were lower and QALY outcomes were higher with the policy, gaining self-reported health benefits).

Results for the CEA, CUA and scenario (lifetime) analysis are reported in table 2; sensitivity analysis results are included in online supplemental appendix 6.

**Table 2:** Results for base case cost-effectiveness, cost-utility and scenario (lifetime) analysis

	Presence of smoke-free policy	Absence of smoke-free policy	Difference Mean (95% CI)	Incremental cost-effectiveness ratio
**1. Cost-effectiveness analysis (incremental cost per 10 µg/m** ^**3**^ **reduction in PM**_**2.5**_ **)**				
People in custody				
Mean cost	£3075	£3142	−£67	Smoke-free policy dominates
Mean PM_2.5_ (10 µg/m^3^)	0.31	3.84	3.53	
Operational staff				
Mean cost	£197	£230	−£33	Smoke-free policy dominates
Mean PM_2.5_ (10 µg/m^3^)	0.31	3.84	3.53	
**2. Cost-utility analysis (incremental cost per quality-adjusted life-year)**				
People in custody				
Mean cost	£3075	£3142	−£67	£1241
Mean QALY	0.682	0.736	−0.054	
Operational staff				
Mean cost	£197	£230	−£33	Smoke-free policy dominates
Mean QALY	0.863	0.859	0.004	
**3. Scenario (lifetime) analysis**				
People in custody				
Mean cost	£22 399	£50 838	−£28 440 (95% CI 29 433 to −27 377)	Smoke-free policy dominates
Mean QALY	21.78	20.81	0.971 (95% CI 0.533 to 1.376)	
Staff				
Mean cost	£12 343	£12 803	−£460 (95% CI −546 to −367)	Smoke-free policy dominates
Mean QALY	29.82	29.55	0.262 (95% CI −0.033 to 0.544)	

dominates—less costly and more beneficial.

PM, particulate matter; QALY, quality-adjusted life-year.

### Scenario (lifetime) analysis

Over a lifetime, the biggest proportion of total costs for the PiC cohort was personal spend on nicotine products; for staff it was morbidity costs (online supplemental appendix 7). For both cohorts, total costs were lower with the policy compared with in the absence of the policy (table 2). This was most marked in the PiC cohort where incremental total costs were −£28 440 (95% CI −£29 433 to −£27 377) compared with −£460 (95% CI −£546 to −£367) in the staff cohort. The number of QALYs for both cohorts was higher with the policy over a lifetime: a difference of 0.971 (95% CI 0.533 to 1.376) for PiC and 0.262 (95% CI −0.033 to 0.544) for staff. These results show that the policy dominates (costs were lower and QALYs were higher) and implementing the policy would be considered cost-effective over a lifetime.

A cost-effectiveness plane, sensitivity analyses results and population-level results are included in online supplemental appendix 7. Sensitivity analyses results showed the policy remains dominant in all analyses. The population-level results indicate combined potential cost savings of more than £200 million to NHS Scotland and on personal nicotine spend over a lifetime, and around 8000 additional QALYs for the total cohort of operational staff and PiC. The share of possible cost savings that relate to NHS Scotland only is around £6 million.

## Discussion

Our data show that the policy is cost-effective in both the short term and long term. Results from the cost-consequence analysis, assessing changes in costs and outcomes over the three phases, showed that while most cost categories decreased, there was no consistent direction of change in outcomes after implementation. The cost-effectiveness analysis, assessing the trade-off of costs against changes in SHS levels in the presence versus absence of the policy, demonstrated cost savings and significant reductions in SHS for PiC and staff. Results from the cost-utility analysis (short term) assessing the trade-off of costs against QALYs in the presence versus absence of the policy demonstrated cost savings and QALY gains for staff, suggesting that the smoke-free policy is optimal for this population. However, the results were less clear for PiC; the cost savings were associated with a reduction in QALYs. Finally, the scenario analysis exploring the potential long-term impact of the policy over a lifetime demonstrated cost-effectiveness for both PiC and staff.

While most cost categories decreased in the short term, some increased; ambulance use, nicotine dependence medication, GP/nurse visits for PiC, PiC e-cigarettes and staff tobacco. In interpreting these results we would expect increases in nicotine dependence medication in preparation for and once the policy was implemented, and PiC e-cigarette costs once tobacco was no longer available on the canteen list. However, it is less clear why the other costs increased. A notable decrease in costs was in smoking-related illness medication, suggesting an association with decreased demand following implementation of the policy. Cost categories driving the decrease in total costs after policy implementation were inpatient stays, smoking-related illness medication and tobacco spend. Key cost categories which increased were mental health stays and e-cigarettes. In interpreting changes in outcomes, decreases in SHS levels and fires were expected with no licit access to tobacco or lighters, but the decrease in PiC health utilities is less easy to interpret. Of the five domains included in the EQ-5D-5L, anxiety/depression showed the biggest decrease for PiC. However, as we do not have identifiable EQ-5D-5L data that can be linked to individuals across phases^26^ (due to the transient nature of the population), the reason behind the decrease in this domain is unclear. Several increasing outcomes (eg, assaults and drug use) included in the analysis are known to be caused by multiple, fluctuating factors, making interpretation of the impact of the policy challenging.^32 33^

Further interpretation of the lifetime sensitivity analyses results demonstrated that the extent to which people resume smoking on release from prison and the cost of tobacco outside prison, were the key drivers of the model results. The population-level extrapolation estimated lifetime cost savings of around £200 million for healthcare and personal nicotine spend among PiC and staff. While we were unable to include full intervention costs, this figure illustrates a substantial cost saving, even if additional intervention costs were incorporated.

This is the first economic evaluation to assess the impacts of implementing a smoke-free policy across an entire national prison service. Research in the prison setting is challenging and often relies on multiple sources of data; we were able to conduct this economic evaluation by incorporating a range of costs and outcome measures. This enabled a comprehensive assessment of the impacts on multiple sectors with an understanding of how and to what extent outcomes changed over time. Furthermore, data from SPS added validity to our findings. While the cost-utility framework is helpful, it is often not sufficient in evaluating public health interventions where not all relevant outcomes are captured by the QALY. By including a cost-consequence analysis we were able to incorporate additional outcomes to show wider impacts of the policy.^34^

Despite being able to amass data on a wide range of pertinent costs and outcomes and to assess benefits and unintended adverse consequences, uncertainty remains relating to routine data identification. As there are no validated methods of identifying healthcare use data for PiC from NHS NSS sources and due to time and other constraints, operationalising individual-level linkage was not possible in this large and transient population. Pragmatic methods of identification were used with input from colleagues from NHS NSS^19^ and this may have affected the precision of the result estimates. Furthermore, data were obtained from several sources in various formats, requiring different methods of analysis, adding to the complexity of the analysis.

Information on the costs of implementing the policy, to the SPS and more widely, was limited. We included the costs of e-cigarette starter kits provided by SPS to eligible PiC in the lifetime analysis, but this alone is likely to underestimate the costs associated with staff training, policy communication, contingency preparation and increased provision of smoking cessation services. Smoking cessation costs were not included because of the differing mechanisms of provision across prisons, although nicotine dependence medication was included and showed an increase in the study period. Costs were not included for monitoring SHS levels inside prisons (which was undertaken as part of the TIPs research^26^) when assessing the need for the policy, as this was considered to be a research cost and not an implementation cost. This is an important consideration for other jurisdictions introducing smoke-free prison policies, as the preannouncement phase SHS exposure measurements partially informed the need for and timing of Scotland’s smoke-free prison policy.^12^ However, costs for equipment, training and adequate SHS measurement are relatively small. We were also unable to include costs such as insurance, air conditioning, building maintenance and restoring fire damage, all of which could arguably have been expected to change as a result of the policy implementation. It is important to note that implementing the policy was achieved reportedly with no major unrest or significant damage to property. It was not possible to include these outcomes in our analyses, although we did include assaults and fires, neither of which saw significant increases. Had major unrest or property damage occurred, the costs to the SPS and beyond could have been significant, as well as causing adverse consequences for PiC and staff.

While evidence to date suggests that e-cigarette use is less harmful than tobacco use,^35^ it is unlikely to prove entirely risk free. Because there is scarce evidence on the effects of long-term e-cigarette use, we did not include any health benefits or harms of e-cigarette use in the lifetime model. Our qualitative research suggests that e-cigarette use behaviours among PiC may differ in some ways to the general population so any benefits or harms may be different in this population. For example, some may be susceptible to heavy use, as a consequence of high nicotine dependence and/or situational factors such as product choice and availability and prison regimes.^23 24^

As this is the first economic evaluation of a smoke-free prison policy, we cannot make direct comparisons to existing research. However, we can compare our model results with what is considered a minimally important QALY gain; 0.074 (95% CI −0.011 to 0.140).^36^ Our individual QALY gains are 0.971 and 0.262 for PiC and staff, respectively, considerably larger than the upper 95% CI of 0.140, so we can assume the model results would be considered important by decision makers. Research on quality of life for hospital employees, comparing an inside-only smoking ban to an inside-and-outside ban, found a lifetime benefit of 0.355 QALYs.^37^ A study evaluating the cost-effectiveness of a complex intervention to reduce SHS exposure in children estimated a cost of £131 per 10 µg/m^3^ reduction in PM_2.5_, compared with absence of policy being dominated in our research.^30^

There is scarce evidence available on the extent to which people resume smoking on release from prison^38^; this a key driver of the long-term results of a smoke-free prison policy evaluation. Future research should monitor rates of resumption on release and consider ways of supporting continued tobacco abstinence, with or without e-cigarettes. Furthermore, research is needed into potential spillover effects on household members when PiC are released. Finally, more evidence is needed on any long-term benefits and harms of e-cigarettes, particularly in this population, to inform decisions about e-cigarette provision by other jurisdictions considering implementing a smoke-free policy in future.

## Conclusion

This study assessed the cost-effectiveness of a smoke-free prison policy compared with no smoke-free policy. The short-term analysis found cost savings for PiC and staff, with no consistent direction of change in outcomes. The lifetime analysis found that implementing the policy was cost-effective for both PiC and staff. In terms of implications, introducing the policy was cost-effective from the traditional cost-utility analysis criteria and when assessing broader outcomes; it was worthwhile adopting the policy. Jurisdictions planning to implement a smoke-free prison policy should consider the provision of e-cigarettes, nicotine dependence medication and smoking cessation support, in light of their national policies, alongside the costs of monitoring SHS levels. The policy was implemented with no major negative impacts. Any future economic evaluations of such policies should consider a broad range of outcomes and ensure the impact on PiC and staff is measured.

**What this paper adds**

**What is already known on this topic**

- In countries, where still permitted, smoking prevalence in prisons is much higher than in the general population, affecting the health of everyone in custody and those working in the prison environment, thus contributing to inequalities in health.
- There is no evidence internationally on the cost-effectiveness of introducing smoke-free policies in a prison setting.

**What this study adds**

- Implementing a smoke-free prison policy is cost-effective over the short term and long term for people in custody and operational staff.

**How this study might affect research, practice or policy**

- As the first economic evaluation of a smoke-free prison policy, this research provides a foundation for methods and evidence in this field internationally.
- Jurisdictions contemplating on implementing a smoke-free prison policy should consider the provision of e-cigarettes, nicotine dependence medication and smoking cessation support.

## Data Availability

No data are available.
