# Supplementary material for: Implementation of a national smoke-free prison policy: an economic evaluation within the Tobacco in Prisons (TIPs) study
Source: Tob Control. 2022 Mar 7;32(6):701–8. doi: 10.1136/tobaccocontrol-2021-056991 (PMC7615232; doi:10.1136/tobaccocontrol-2021-056991)
Supplement: Supplementary data [file tobaccocontrol-2021-056991supp001.pdf]

Supplementary Appendix

Appendix 1 Resources included in short-term base-case analyses

Supplementary Table 1 Data (PiC) available separately for closed and open prisons in base-case analysis

**Resource/Outcome**	**Data available separately for closed and open prisons – therefore open prison excluded from analysis**
GP/nurse - PiC	✓
Outpatient/inpatient/mental health stay	
Ambulance	✓
A&E	
Medication	✓
Staff tobacco	
Pic tobacco and NVP	✓
QALYs - PiC	✓
Violence	✓
Deaths	
Fires	✓
MoRs	✓

Appendix 2 Short-term analyses: sources, format and unit costs for resources use data

Identifying resource use

Health service use

GP and nurse visits (PiC and staff)

Data were extracted from TIPs staff and PiC surveys in all phases for the number of visits to GP/nurse reported in the previous 3 months, from which monthly resource use was calculated.

Outpatient attendances, inpatient and mental health length of stay, and accident and emergency attendances (PiC only)

These data were provided by NHS National Services Scotland (NHS NSS). The conventional method of identifying individual patient resource use in this data is the Community Health Index (CHI) number, a unique identifier given to patients accessing Scottish health care. Due to the complexity and practicalities of linking individual records from the above datasets, other identification methods for PiC health service use were applied. These are described for each dataset below. All data extracted covered the study period of 1 June 2016 to 30 November 2019 and were received in an aggregate monthly format.

Outpatient attendances (SMR00)

Two variables available in the outpatient dataset were used to identify PiC attendances: 1) GP practice code (prison GP practices are all allocated the same code), and 2) 'referral source code', for these purposes the referral category 'prison/penal establishment' was used. Data identified by at least one of these variables were included in the analysis.

Inpatient and mental health length of stay (SMR01 and SMR04)

Two variables, GP practice code and 'admission/transfer from' were used to identify PiC resource use in the inpatient and mental health datasets for length of stay, the admission category 'legal establishment including prison' was used. Data identified by at least one of these variables were included in the analysis.

Accident and emergency attendances (A&E2)

Accident and emergency attendances were identified using the 'postcode of residence' field, this was feasible as prisons have unique postcodes.

Ambulance incidents (PiC only)

The aggregate number of ambulance incidents was provided by SPS for each prison, from 1 June 2016 to 30 November 2019.

Medication (PiC only)

Medication dispensed to PiC from 1 June 2016 to 30 November 2019 was provided by National Procurement, NHS NSS. Data was included for named patients and stock supply, extracted as an aggregate monthly cost. The base-case analysis includes two categories of medication; 1) indicators of nicotine dependence and 2) indicators of smoking-related illnesses or associated symptoms, the rationale for this is these are the medications most likely to change following the implementation of a smoke-free policy. A sensitivity analysis (SA1) was conducted including all medication.

Nicotine products

PiC tobacco and NPV

PiC spending on tobacco and NPV products was extracted from canteen data provided by SPS for a TIPs related CRUK-funded study. Each prison has a canteen (shop) from which PiC can purchase items on the canteen list; tobacco products were taken off the canteen list in November 2018 and NPV were available on the canteen list from September 2018. Data was available from 29 July 2018 for both tobacco and NPV (four months prior to implementation of the smoke-free policy), to November 2018 for tobacco and November 2019 (end of study period) for NPV. Analysis showed that spend on tobacco products was constant in the months leading to the policy implementation and we assumed that tobacco spend earlier than 29 July 2018 was consistent with the spend from July 2018 onwards, an average monthly spend was applied to the earlier months of the study period based on mean spend for August to October 2018 when full months' data was available. Data was not available for Scotland's two privately run prisons.

Staff tobacco

The weekly number of cigarettes smoked was extracted from the TIPS staff surveys from each phase combining the reported number of cigarettes smoked on workdays and days off. The weekly consumption of cigarettes was assumed to be constant across each phase and a monthly usage was estimated based on this assumption, for each phase.

Supplementary Table 2 Short-term analyses: resource use sources and formats summary

	**Source**	**Time period data covers**	**Data point frequency**	**Data format**
**Resource use**				
**Healthcare**				
People in custody				
GP/nurse visits	Survey - number of visits in previous 3 months	All 3 phases	3 timepoints; Nov 2016/April 2017, June/July 2018 & May/June 2019	From individuals, (mean, sd n)
Outpatients	NHS NSS (ISD SMR00)	Jun 2016 -Nov 2019	Monthly	Aggregate data for all PiC, (total monthly count)
Inpatient stays	NHS NSS (ISD SMR01)	June 2016 to Nov 2019	Monthly	Aggregate data for all PiC, (total monthly count – admissions and los)
Mental health stays	NHS NSS (ISD SMR04)	June 2016 to Nov 2019	Monthly	Aggregate data for all PiC, (total monthly count – admissions and los)
A&E	NHS NSS (ISD Unscheduled Care A&E2)	June 2016 to Nov 2019	Monthly	Aggregate data for all PiC, (total monthly count)
Ambulance	Scottish Prison Service	June 2016 to Nov 2019	Monthly	Aggregate data for all PiC, (total monthly count)
Medication	National Procurement, NHS NSS	June 2016 to Nov 2019	Weekly	Aggregate data for all PiC, (total monthly count)
Staff				
GP visits	Staff survey - number of visits in previous 3 months	All 3 phases	3 timepoints; Nov/Dec 2016, May/July 2018 & April/July 2019	From individuals, (mean, sd n)
**Nicotine products**				
People in custody				
Cigarettes	SPS canteen data	3 months prior to ban	Monthly	Aggregate data for all PiC (total

				resource use and spend)
Nicotine vaping products	SPS canteen data	3 months prior to ban, onwards	Monthly	Aggregate data for all PiC (total resource use and spend)
Staff				
Cigarettes	Survey – phase 1, 2 & 3, how many cigarettes per day	All 3 phases	3 timepoints; Nov/Dec 2016, May/July 2018 & April/July 2019	From individuals, (mean, sd n)

Supplementary Table 3 Short-term analyses: unit costs

Resource use	Unit cost	Source
**Health**		
People in custody		
GP/nurse visits	£20.30	PSSRU 2017/18 GP and nurse, length of consultation from PSSRU 2015
Outpatients	£176.00	ISD cost book 2017/18 R044X, consultant led new patient, cost per attendance
Inpatient stays		
Mean across all specialities	£1,190.00	ISD cost book 2017/18 R040, cost per bed day
Accident & Emergency	£1,492.34	
Cardiac Surgery	£2,042.72	
Cardiology	£720.16	
Clinical Oncology	£1,009.80	
Dermatology	£514.63	
Ear, Nose & Throat	£1,290.04	
Gastroenterology	£554.54	
General Medicine	£482.35	
General Surgery (exc Vascular Surgery)	£865.83	
Gynaecology	£1,543.31	
Haematology	£1,097.21	
Intensive Care Unit	£2,217.48	
Medical Oncology	£1,286.00	
Neurology	£1,128.05	
Neurosurgery	£1,439.17	
Ophthalmology	£1,596.58	
Plastic Surgery & Burns	£1,705.61	
Respiratory Medicine	£510.46	
Thoracic Surgery	£1,359.73	
Urology	£938.50	

Vascular Surgery	£652.04	
Mental health stays		
General Psychiatry	£3,645	ISD cost book R040LS, cost per inpatient week
Geriatric Psychiatry	£2,669	
Learning Disabilities	£4,833	
Adolescent Psychiatry	£6,158	
Child and Adolescent Psychiatry	£6,158	
A&E	£137	ISD cost book 2017/18, R044 cost per attendance
Ambulance	£310	ISD cost book R910 2017/18 cost per incident (accident & emergency, All Scotland)
Medication	Various	Included in National Procurement dataset
Staff		
GP visits (staff	£31.30	PSSRU 2017/18
**Personal**		
People in custody		
Cigarettes	Various	Included in SPS canteen data
E-cigs/vapes	Various	Included in SPS canteen data
Staff		
Cigarettes	£10.23 for 20 king size	ONS

Appendix 3 Short-term analyses: sources and format for outcome data

Supplementary Table *4* Short-term analyses: outcomes sources and formats

**Outcomes**				
	**Source**	**Time period data covers**	**Data point frequency**	**Data format**
Utilities	TIPS staff and PiC surveys	All 3 phases	3 timepoints, one in each phase	From individuals, (mean, sd n)
Concentration of second-hand smoke levels ($PM_{2.5}$)	TIPs study	All 3 phases	3 timepoints, one in each phase	Per prison (1, 2)
Prisoner on staff assaults	SPS	November 2017 to November 2019	Monthly	Aggregate data for all PiC (total count)
'Prisoner-on-prisoner' assaults	SPS	November 2017 to November 2019	Monthly	Aggregate data for all PiC (total count)
All-cause mortality (deaths in	SPS	June 2016 to November	Monthly	Aggregate data for all PiC (total count)

custody) amongst PiC		2019		
Fires	SPS	June 2016 to November 2019	Monthly	Aggregate data for all PiC (total count)
The Management of an Offender at Risk due to any Substance Policy (MORS)	SPS	June 2016 to November 2019	Monthly	Aggregate data for all PiC (total count)

PiC – people in custody

Appendix 4 Scenario (lifetime) analysis : description and model input parameters

Model structure

The model is split into two time periods; 'in prison' and 'post-prison', each with four states ('Tobacco smoker', 'Quit/tobacco abstinent', 'Non-tobacco smoker' and 'Death'). PiC enter the model at the start of their first custodial sentence and staff enter the model when they begin employment with SPS. PiC enter the post-prison period on release from prison and staff on leaving employment with SPS.

Within each time period participants can either remain in their state, transition from 'Tobacco smoker' to 'Quit/tobacco abstinent', or transition from any live state to 'Death'. When transitioning from the 'in prison' to the 'post-prison' period, model participants can remain in the state they were previously in; this is always the case with participants in the 'Non-tobacco smoker' state. 'Tobacco smokers' can also transition to 'Quit/tobacco abstinent', and 'Quit/tobacco abstinent' can resume smoking. Following the implementation of smoke-free prison policy, all PiC entering the model as 'tobacco smokers' will immediately transition to 'Quit/tobacco abstinent'.

The following model assumptions were applied:

- All participants in the 'Non-tobacco smoker' state in the 'in prison' period are assumed to be exposed to SHS for the purposes of morbidity, mortality and health utilities in the pre-policy comparator.
- Participants in the 'Non-tobacco smoker' state 'post-prison' are assumed not to be exposed to SHS.
- Participants in the 'Non-tobacco smoker' state are assigned never-smoker morbidity, mortality and health utilities; there is no distinction between former smokers and never smokers.
- All participants in the 'Quit/tobacco abstinent' state are assigned former smoker morbidity, mortality and health utilities, in line with existing economic smoking cessation models (3).
- Non-tobacco smokers do not start smoking at any time during the model; this assumption is mitigated by applying PiC smoking prevalence (4).

Input parameters

For the base-case analysis, where possible, parameters were sourced from TIPs surveys and SPS reports and information. Where there were gaps in the evidence other sources were used, including literature (referenced below) and expert opinion.

Model input parameters are reported in Supplementary Table 5 and described below.

Model transitions

Smoking status

Smoking prevalence for PiC and staff were sourced from the Phase-1 TIPs survey (5) and used to populate the initial states in the 'in prison' period in both comparators. PiC entered the model as either 'Tobacco smokers' or 'Non-tobacco smokers' pre-policy, and 'Quit/tobacco abstinent' or 'Non-tobacco smokers' post-policy. Staff were either 'Tobacco smokers' or 'Non-tobacco smokers' in both comparators.

To account for PiC and staff who quit smoking in the model, a background quit rate was applied to the 'Tobacco smoker' state, in line with existing smoking cessation models (6).

For PiC entering the 'post-prison' period in the post-policy comparator assumptions about smoking resumption were sourced from the PiC TIPs survey on perception of smoking status post-release from prison.

Morbidity

Smoking related morbidity was applied to all model participants: diseases comprised coronary heart disease (CHD), chronic obstructive pulmonary disease (COPD), lung cancer and stroke (7). Morbidity transitions were specific to smoking status, gender and age and used three inputs; sex and age specific morbidity prevalence irrespective of smoking behaviour; the prevalence of sex and age specific smoking behaviour; and the sex and age specific morbidity relative risk for current and former smokers, with never smokers assigned a relative risk of 1. The prevalence of each smoking related disease was assumed to be independent of others given the lack of evidence to support alternative assumptions.

There is evidence of a causal link between exposure to SHS and CHD and lung cancer in never smokers (8), so non-tobacco smoking model participants pre-policy (in prison) are assigned CHD and lung cancer morbidity adjusted for exposure to SHS.

Mortality

Sex, age and smoking status specific mortality rates were applied based on the general Scottish population data and calculated as follows. Scottish sex and age specific mortality rates for 2018 (9) were modified for the increased risk of mortality for smokers and former smokers reported by Doll et al (10), and sex and age specific smoking prevalence for the Scottish general population was applied to this calculation (11).

There is evidence to suggest that PiC have a lower mortality rate while in prison and a higher mortality rate when released from prison compared to the general population (12-14). To adjust for these different mortality rates, sex specific standardised mortality ratios were applied to the mortality rates for the general Scottish population. The general Scottish population mortality rates were applied to staff with no adjustment.

It is estimated that exposure to SHS accounts for 1% of total mortality globally (8, 15): to replicate this increased mortality for non-tobacco smoking model participants, the mortality of those not exposed to SHS was reduced by 1%. These model participants comprised non-tobacco smokers in the 'post-prison' period for both comparators and non-tobacco smokers 'in prison' post-policy.

Costs

Resource use categories comprised the intervention, annual healthcare for morbidities, and spend on nicotine products by PiC and staff, valued at 2017/18 prices, uplifted where needed (16). Sources include a freedom of information request (FOI), literature, SPS canteen data analysed in a parallel CRUK-funded study and the TIPs surveys.

Intervention costs

Intervention costs comprise the cost to the SPS of providing e-cigarettes starter packs to PiC (17). This cost was applied to all PiC post-policy regardless of smoking status in the model as a conservative approach.

Healthcare costs

The annual costs of CHD, COPD, lung cancer and stroke were applied to model participants with each morbidity (3).

Tobacco and nicotine vaping products

The mean weekly spend on tobacco by PiC was extracted from SPS canteen data and was assumed to be consistent throughout the year. An annual cost was estimated based on this and applied to PiC in the 'Tobacco smoker' state in the 'in prison' period pre-policy. Post-policy no tobacco costs for PiC were applied in the 'in prison' period. In the 'post-prison' period for both comparators the reported daily number of cigarettes smoked by PiC was extracted from the Phase-1 TIPs survey. The usage was assumed to be consistent across the year to allow estimation of an annual usage, and a cost per cigarette applied to this total. Weekly e-cigarette costs for the 'in prison' period post-policy were extracted from SPS canteen data. E-cigarette costs were assumed to be consistent throughout the year and an annual cost was calculated. This annual cost was applied to all former smokers in the 'in prison' period post-policy.

The number of reported daily cigarettes smoked by operational staff was extracted from the TIPs survey for Phases I and III, to represent the without and with a smoke-free policy comparators. An annual usage was calculated assuming that this reported number of cigarettes smoked was consistent throughout the Phases and year and a cost per cigarette applied to the annual usage.

Outcomes

The model outcome was the QALY, a combination of quality and length of life. A quality of life utility was estimated using responses to the EQ-5D-5L questionnaires in the TIPs surveys, dependent on smoking status, for PiC and staff, for Phase-1 and Phase-3. Non-smoker health utilities reported in Phase-1 of the TIPs surveys were assumed to include a decrement related to SHS exposure. Model participants allocated a smoking related morbidity were assigned a disease specific health utility sourced from literature, as is best practice (18).

Other model inputs

The mean age entering the model was 27 years for PiC (12) and 28 years for operational staff (data provided by SPS and based on the position at 31st March 2020).

The mean length of time in the 'in-prison' period was three years for PiC (calculated using the mean length of sentence and mean number of incarcerations in Scotland (12, 19, 20)), and 16 years for staff (provided by SPS and based on the position at 31st March 2020).

Analysis

The model was developed using Microsoft Excel for Office 365. Results from the sex specific cohorts were combined applying ratios of male to female PiC (95:5) and staff (73:27) to estimate a per person cost and QALY. Base-case results presented for PiC and staff include a breakdown of total mean costs, total and incremental mean costs and QALYs and 95% confidence intervals around incremental results.

Uncertainty

Uncertainty in input parameters was characterised with a probabilistic sensitivity analysis (PSA) using best practice techniques (21), with results used to estimate the 95% confidence interval around the costs, QALYs and the ICER. The following distributions were fitted for the PSA: for relative risk, lognormal; for costs, gamma and for health utilities, beta. 1,000 iterations were run based on model convergence guidance (22) and results were plotted on a cost-effectiveness plane to visually represent uncertainty.

Several plausible sensitivity analyses were conducted to assess the impact of varying input parameters on the base-case cost-effectiveness results, altering the length in prison, resumption of tobacco smoking on release, varying PiC spend on nicotine products, applying health utilities from the literature and replacing TIPs input parameters with parameters from the literature as far as possible. Input parameters for the sensitivity analysis are included in Supplementary Table 6.

Population-based analysis

For the population-based analysis the mean number of PiC was 7,500 (based on mean daily population SPS annual report and accounts 2017-18 (23)), and the number of staff was 3,244 (provided by SPS and based on the position at 31st March 2020). In this analysis the total cost of vaping kits to SPS was applied for intervention costs(24).

Supplementary Table 5 Scenario (lifetime) analysis: lifetime model base-case input parameters

Parameter	Mean	PSA – distribution (alpha, beta)	Source
**Model transitions**			
Smoking prevalence:			
PiC	0.74	N/A	TIPs PiC survey
Staff	0.09	N/A	TIPs staff survey
Background quit rate	0.02	N/A	NICE(12)
Resumption on release (PiC)	0.42	N/A	TIPs PiC survey
Smoking related morbidity	Various depending on disease, age and sex	Lognormal (various depending on disease, age and sex)	Jones et al.(3)
Morbidity SHS exposure:			

CHD	1.27 (1.19-1.36)	Lognormal (lnmean 0.239, lnse 0.034)	Öberg et al.(25)
Lung cancer	1.21 (1.13–1.30)	Lognormal (lnmean 0.191, lnse 0.036)	
Mortality:			
PiC mortality SMR:			Graham et al.(12)
In prison – female PiC	1.9 95% CI 0.9, 3.5	Lognormal (lnmean 0.642, lnse 0.346)	
In prison – male PiC	0.6 95% CI 0.5, 0.7	Lognormal (lnmean -0.511, lnse 0.086)	
Out of prison – female PiC	5.9 95% CI 5.4, 6.5	Lognormal (lnmean 1.775, lnse 0.0.047)	
Out of prison -male PIC	2.5 95% CI 2.4, 2.6	Lognormal (lnmean 0.916, lnse 0.020)	
Staff mortality	Various depending on age, sex and smoking status	Lognormal (various depending on disease, age and sex)	National Records of Scotland(26), Doll et al.(27) & Scottish Health Survey 2017(28)
**Costs:**			
Intervention (per person)	£14 per PiC	N/A	BBC FOI(24)
Intervention (population level)	£150,000 for whole population	N/A	BBC FOI(24)
Morbidity healthcare:			
CHD	£1,958 (SE £195)	Gamma (99.95, 19.59)	Jones et al(3)
COPD	£899 (SE £90)	Gamma (100.07, 8.98)	
Lung cancer	£10,178 (SE £1,018)	Gamma (100.00, 101.78)	
Stroke	£4,630 (SE £32)	Gamma (21578.7, 0.215)	
Nicotine personal spend:			
Mean price of 20 king size filter cigarettes (2018)	£10.23	N/A	ONS(29)
Staff tobacco (absence of smoke-free policy)	£2,290 (SE 20.64)	Gamma (12312.9, 0.186)	ONS(29) applied to TIPs staff questionnaire (phase 1)
Staff tobacco (presence of smoke-free policy)	£2,054 (SE 23.65)	Gamma (7538.7, 0.272)	ONS(29) applied to TIPs staff questionnaire (phase 3)
PiC Tobacco (absence of smoke-free policy) 'in prison'	£412 (SE 0.665)	Gamma (384014.3, 0.001)	Canteen data
PiC Tobacco (both comparators) 'post-prison'	£3,590 (SE 29.80)	Gamma (14511.1, 0.247)	ONS(29) applied to TIPs PiC questionnaire (Phase 1)
PiC NVP (presence of smoke-free policy)	£306 (SE 0.399)	Gamma (586379.6, 0.0005)	Canteen data
**Outcomes - utilities:**			
Staff			
Never smoker (absence of smoke-free policy)	0.854	Beta (3309.37, 565.77)	TIPs staff questionnaire Phase 1

Former smoker (absence of smoke-free policy)	0.863	Beta (3326.49, 528.08)	TIPs staff questionnaire Phase 1
Current smoker (absence of smoke-free policy)	0.87	Beta (536.99, 80.24)	TIPs staff questionnaire Phase 1
Never smoker (presence of smoke-free policy)	0.874	Beta (1082.84, 156.11)	TIPs staff questionnaire Phase 3
Former smoker (presence of smoke-free policy)	0.848	Beta (1244.09, 223.0)	TIPs staff questionnaire Phase 3
Current smoker (presence of smoke-free policy)	0.882	Beta (283.62, 37.95)	TIPs staff questionnaire Phase 3
PiC			
Never smoker (absence of smoke-free policy)	0.777	Beta (795.86, 228.41)	TIPs PiC questionnaire Phase 1
Former smoker (absence of smoke-free policy)	0.778	Beta (932.37, 266.05)	TIPs PiC questionnaire Phase 1
Current smoker (absence of smoke-free policy)	0.722	Beta (4024.75, 1549.70)	TIPs PiC questionnaire Phase 1
Never smoker (presence of smoke-free policy)	0.779	Beta (463.28, 131.43)	TIPs PiC questionnaire Phase 3
Former smoker (presence of smoke-free policy)	0.775	Beta (1116.09, 324.03)	TIPs PiC questionnaire Phase 2
Current smoker (presence of smoke-free policy)	0.708	Beta (2986.42, 1231.69)	TIPs PiC questionnaire Phase 2

CHD – coronary heart disease; CI – confidence interval; COPD – chronic obstructive pulmonary disease; lnmean – lognormal of mean; lnse – lognormal of standard error; PiC – person/people in custody; PSA – probabilistic sensitivity analysis; SHS – second-hand smoke; SMR – standardised mortality ratio

Supplementary Table 6 Scenario (lifetime) analysis: sensitivity analyses input parameters

**Scenario**	**Base-case assumptions**	**Input parameters**	**Source**
1) PiC resumption of smoking on release	42% resumption based on TIPs PiC survey results	0.95	Plausible level of resumption (30)
2) PiC time in prison – 1 year	3 years based on mean length of sentence and	1 year	Plausible alternative to demonstrate effect of length of sentence on results
3) PiC time in prison – 5 year	3 years based on mean length of sentence and	5 years	
4) PiC 'In prison' tobacco and e-cigarette spend: TIPs survey	PiC 'In prison' tobacco and e-cigarette spend based on canteen data	£693	TIPs PiC survey
5) PiC 'Post prison' tobacco spend: canteen 'in prison' data	Based on TIPs PiC reported number of cigarettes smoked plus ONS unit cost of cigarette	£412	Canteen data

6) Published utilities (PiC and staff)	Utilities reported in TIPs PiC and staff surveys	Never smoker exposed to SHS (1.1% decrement)	0.869	Maheswaran H., Petrou S., Rees K., Stranges S. Estimating EQ-5D utility values for major health behavioural risk factors in England. J Epidemiol Community Health 2013; 67: 172–80
		Never smoker – no SHS	0.879	
		Former smoker	0.818	
		Moderate smoker	0.828	
		Heavy smoker	0.767	
7) Replacing TIPs data with published data as far as possible				
PiC smoking prevalence	74% TIPs PiC survey	68%		SPS 16th Prisoner survey
Staff smoking prevalence	9% TIPs staff survey	20% male and 16% female		Scottish Health Survey
PiC resumption of smoking on release	42% TIPs PiC survey	100%		Most conservative scenario – all PiC resume tobacco smoking on release
Utilities	TIPs PiC survey	Never smoker exposed to SHS (1.1% decrement)	0.869	Maheswaran H., Petrou S., Rees K., Stranges S. Estimating EQ-5D utility values for major health behavioural risk factors in England. J Epidemiol Community Health 2013; 67: 172–80
		Never smoker – no SHS	0.879	
		Former smoker	0.818	
		Moderate smoker	0.828	
		Heavy smoker	0.767	

Appendix 5 Short-term cost-consequence analysis results

Supplementary Table 7 Cost-consequence analysis results: changes between phases (base-case analysis)

	Change Phase 1 v Phase 2		Change Phase 2 v Phase 3	
	Step change	Slope change	Step change	Slope change
	Co-efficient (95% CI)	Co-efficient (95% CI)	Co-efficient (95% CI)	Co-efficient (95% CI)
**Base-case - Costs**				
**Outpatient ǂ**	-0.731 (-1.64 to 0.173)	-0.003 (-0.093 to 0.086)	-0.609 (-0.875 to -0.343)	-0.004 (-0.056 to 0.048)
**Inpatient ǂ**	-8.56 (-14.9 to -2.23)	0.110 (-0.498 to 0.719)	0.443 (-6.93 to 7.81)	-1.13 (-1.85 to -0.409)
**Mental health hospital stays ǂ**	-193 (-729 to 342)	-62.7 (-140 to 15.0)	-266 (-497 to -35.3)	4.96 (-25.4 to 35.3)
**Accident & emergency visits ǂ**	0.045 (-0.146 to 0.236)	-0.037 (-0.065 to -0.010)	-0.061 (-0.206 to 0.083)	-0.047 (-0.080 to -0.013)
**Ambulance ǂ**	0.330 (-0.081 to 0.740)	-0.061 (-0.108 to -0.014)	0.340 (-0.084 to 0.764)	0.025 (-0.045 to 0.094)
**Medication (nicotine dependence) ǂ**	-0.194 (-0.592	0.049 (-0.015 to	-0.578 (-1.73 to	-0.032 (-0.189

	to 0.205)	0.113)	0.574)	to 0.124)
**Medication (smoking related illness) ǂ**	-3.59 (-10.4 to 3.25)	-0.337 (-1.01 to 0.339)	-0.233 (-0.723 to 0.257)	0.008 (-0.029 to 0.044)
**E-cigarettes ǂ**	N/A	N/A	11.3 (9.33 to 13.3)	-0.741 (-1.33 to -0.155)
	**Change Phase 1 v Phase 2**		**Change Phase 2 v Phase 3**	
**Person in custody GP/nurse visits ***	0.677 (-0.399 to 1.75)		0.447 (-1.32 to 2.22)	
**Staff GP visits ***	-0.364 (-1.07 to 0.345)		-0.675 (-1.45 to 0.099)	
**Person in custody tobacco ***	N/A		N/A	
**Staff tobacco ***	0.049 (-5.34 to 5.44)		-0.979 (-7.19 to 5.23)	
**Base-case - Outcomes**				
	**Step change**	**Slope change**	**Step change**	**Slope change**
	**Co-efficient (95% CI)**	**Co-efficient (95% CI)**	**Co-efficient (95% CI)**	**Co-efficient (95% CI)**
**Prisoner-on-staff assaults**	N/A	N/A	-0.001 (-0.003 to 0.001)	-0.0001 (-0.0004 to 0.0002)
**Prisoner-on-prisoner assaults**	N/A	N/A	0.004 (-0.001 to 0.009)	-0.0008 (-0.002 to 0.0001)
**All cause mortality (deaths in custody)**	-0.00003 (-0.0003 to 0.0003)	0.00002 (-0.00002 to 0.00005)	0.0002 (-0.0001 to 0.0005)	0.000002 (-0.00005 to 0.00004)
**Fires**	0.0005 (0.00002 to 0.001)	0.00004 (-0.00004 to 0.0001)	-0.0009 (-0.002 to -0.00002)	-0.00005 (-0.0001 to 0.00005)
**MORS**	0.009 (0.0006 to 0.017)	0.00005 (-0.0008 to 0.0009)	0.003 (-0.007 to 0.013)	-0.0002 (-0.001 to 0.001)
	**Change Phase 1 v Phase 2**		**Change Phase 2 v Phase 3**	
**Levels of second-hand smoke (µg/m³) Mean $PM_{2.5}$ (SD) ***	-27.3 (-43.8 to -10.8)		-8.01 (-10.5 to -5.6)	
**Utilities (person in custody) Mean (SD) ***	-0.010 (-0.026 to 0.005)		-0.043 (-0.062 to -0.025)	
**Utilities (staff) Mean (SD) ***	0.003 (-0.008 to 0.014)		0.001 (-0.014 to 0.015)	

ǂ time series analysis, * regression framework, MORS - The Management of an Offender at Risk due to any Substance

Supplementary Table 8 Cost-consequence results: changes between phases (sensitivity analyses)

	**Change Phase 1 v Phase 2**		**Change Phase 2 v Phase 3**	
	**Step change**	**Slope change**	**Step change**	**Slope change**
	**Co-efficient (95% CI)**	**Co-efficient (95% CI)**	**Co-efficient (95% CI)**	**Co-efficient (95% CI)**
**Sensitivity analysis 1 Costs - all medication**				
**Medication (all) ǂ**	14.2 (-12.7 to 41.0)	-1.10 (-3.58 to 1.37)	2.58 (-17.0 to 22.1)	0.280 (-2.98 to 3.54)
**Sensitivity analysis 2 Costs - including open prison**				
**Ambulance ǂ**	0.269 (-0.177	-0.059 (-0.111 to -	0.325 (-0.071	0.020 (-0.046 to

	to 0.716)	0.007)	to 0.720)	0.085)
**Medication (nicotine dependence) ǂ**	-0.209 (-0.651 to 0.233)	0.037 (-0.030 to 0.104)	-0.435 (-1.62 to 0.747)	-0.031 (-0.198 to 0.136)
**Medication (smoking related illness) ǂ**	-3.54 (-10.3 to 3.25)	-0.340 (-1.00 to 0.321)	-0.225 (-0.707 to 0.256)	0.007 (-0.038 to 0.052)
**Medication (all) ǂ**	12.9 (-12.3 to 38.1)	-1.35 (-3.75 to 1.06)	1.30 (-16.9 to 19.6)	0.072 (-3.03 to 3.17)
**E-cigarettes ǂ**	N/A	N/A	11.1 (9.18 to 13.0)	-0.772 (-1.32 to -0.224)
	**Change Phase 1 v Phase 2**		**Change Phase 2 v Phase 3**	
**GP/nurse visits***	0.619 (-0.438 to 1.68)		0.481 (-1.24 to 2.20)	
**Sensitivity analysis 2 Outcomes – including open prison**				
	**Step change**	**Slope change**	**Step change**	**Slope change**
	**Co-efficient (95% CI)**	**Co-efficient (95% CI)**	**Co-efficient (95% CI)**	**Co-efficient (95% CI)**
**Prisoner-on-staff assaults ǂ**	N/A	N/A	-0.001 (-0.003 to 0.001)	-0.0001 (-0.0004 to 0.0002)
**Prisoner-on-prisoner assaults ǂ**	N/A	N/A	0.004 (-0.001 to 0.009)	-0.0008 (-0.002 to 0.00005)
**Fires ǂ**	0.0005 (0.00004 to 0.001)	0.00003(-0.00004 to 0.0001)	-0.009 (-0.002 to -0.000008)	-0.00004 (-0.0001 to 0.00007)
**MORS ǂ**	0.009 (0.0008 to 0.016)	0.0002 (-0.0006 to 0.001)	0.002 (-0.008 to 0.013)	-0.0001 (-0.001 to 0.001)
	**Change Phase 1 v Phase 2**			**Change Phase 2 v Phase 3**
**Utilities Mean (SD) ***	-0.007 (-0.022 to 0.008)		-0.044 (-0.063 to -0.026)	

ǂ time series analysis, * regression framework, MORS - The Management of an Offender at Risk due to any Substance

Appendix 6 Short-term cost-effectiveness and cost-utility analyses results

Supplementary Table 9 Cost-effectiveness and cost-utility analyses: costs breakdown

**Resource**	**Presence of smoke-free policy £**	**Absence of smoke-free policy £**
**Base-case**		
People in custody		
Health services resources		
GP/nurse visits	57	44
Inpatient	93	122
Outpatient	30	45
Mental health stays	2,599	2,548
A&E visits	18	18
Ambulance	16	8

Medication – smoking related illness	9	34
Medication – nicotine dependence	34	21
Personal		
Tobacco	-	302
E-cigarettes	219	-
**Total for PiC**	**3,075**	**3,142**
Operational staff		
GP visits	24	36
Tobacco	173	194
**Total for staff**	**197**	**230**
**Sensitivity analysis 1 – all medication (PiC)**		
Health services resources		
GP/nurse visits	57	44
Inpatient	92	122
Outpatient	30	45
Mental health stays	2,599	2,548
A&E visits	18	18
Ambulance	16	8
Medication – all	1,696	1,108
Personal		
Tobacco	-	302
E-cigarettes	219	-
**Total**	**4,727**	**4,195**
**Sensitivity analysis 2 – including open prison (PiC)**		
Health services resources		
GP/nurse visits	57	43
Inpatient	92	122
Outpatient	30	45
Mental health stays	2,599	2,548
A&E visits	18	18
Ambulance	16	9
Medication – smoking related illness	9	33
Medication – nicotine dependence	35	22
Personal		
Tobacco	-	299
E-cigarettes	216	-
**Total**	**3,072**	**3,139**

Supplementary Table 10 Cost-effectiveness and cost-utility analyses: sensitivity analysis

	**Presence of smoke-free policy**	**Absence of smoke-free policy**	**Difference**	**Cost-effectiveness**

**Cost-effectiveness analysis (Incremental cost per 10µg/m^3 reduction in $PM_{2.5}$)**				
**Sensitivity analysis - all dispensed medication (person in custody)**				
**Person in custody**				
Mean cost	£4,727	£4,195	£532	£151 per 10µg/m^3 reduction in $PM_{2.5}$
Mean $PM_{2.5}$ (10µg/m^3)	0.31	3.84	3.53	
**Sensitivity analysis - including open prison data (person in custody)**				
**Person in custody**				
Mean cost	£3,072	£3,139	-£67	Smoke-free policy dominates
Mean $PM_{2.5}$ (10µg/m^3)	0.31	3.84	3.53	
**Cost-utility analysis (Incremental cost per quality adjusted life-year)**				
**Sensitivity analysis - all dispensed medication (person in custody)**				
**Person in custody**				
Mean cost	£4,727	£4,195	£532	Absence of smoke-free policy dominates
Mean quality adjusted life-year	0.682	0.736	-0.054	
**Sensitivity analysis - including open prison data (person in custody)**				
**Person in custody**				
Mean cost	£3,072	£3,139	-£67	£1,241 per quality adjusted life-year
Mean quality adjusted life-year	0.685	0.736	-0.051	

Appendix 7 Scenario (lifetime) analysis

Table 11 Scenario (lifetime) analysis: costs breakdown

People in custody		
**Costs**		
Implementation	£14	£0
Morbidity	£6,308	£7,079
Personal	£16,077	£43,759
**Total costs**	£22,399	£50,838
Staff		
**Costs**		
Implementation	£0	£0
Morbidity	£8,325	£8,337
Personal	£4,019	£4,467
**Total costs**	£12,343	£12,803

CI – confidence interval

Uncertainty is illustrated with results from the probabilistic sensitivity analysis plotted onto a cost-effectiveness plane (Supplementary Figure 1). This figure shows that there was minimal uncertainty in costs but more uncertainty in QALYs. It also shows that all plots for the PiC cohort demonstrate dominance of the smoke-free policy. In the staff cohort this uncertainty in QALY gains is illustrated by the plots crossing the vertical axis: a small proportion of the incremental QALYs fall to the left of the vertical axis (<1%), however, overall, there was an overall mean QALY gain.

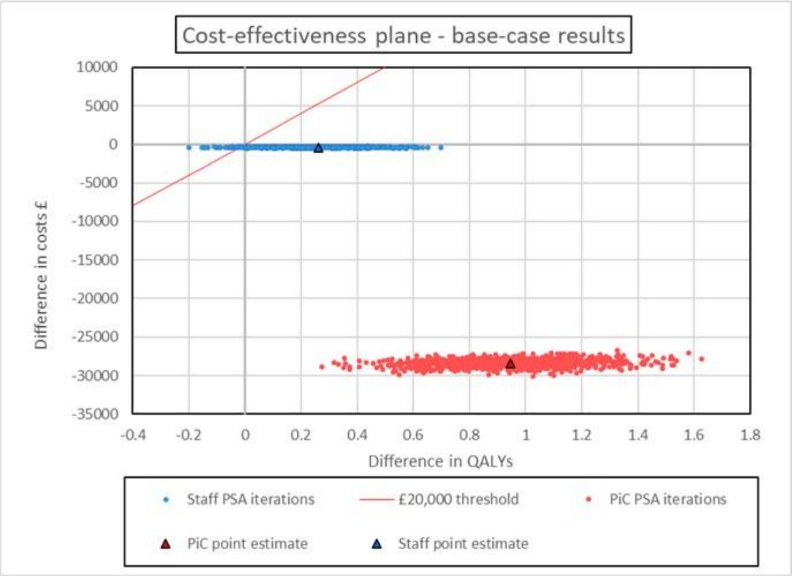

Supplementary Figure 1 Scenario (lifetime) analysis: cost-effectiveness plane

Sensitivity analyses results

In all sensitivity analyses, the smoke-free policy dominates, confirming the robustness of the base-case results. Full results are reported in Supplementary Table 10 and ICERs are illustrated Supplementary Figure 2.

The analyses with the biggest impact on the ICER for PiC were Scenario 5 (*'Post-prison' tobacco spend: canteen 'in prison' data)* and Scenario 7 *(Replacing TIPs data with published data as far as possible).* These two scenarios reduce the ICER, although 'with a smoke-free policy' still dominates. In Scenario 5 the impact on the ICER is driven by the reduction in tobacco costs in the 'post-prison' period; in the base-case analysis tobacco costs after release from prison are based on the Office for National Statistics (ONS) cost which is higher than the cost of tobacco on sale in prison; taking account of this difference reduces the ICER from -£21,127 to -£3,013. Scenario 7 is driven by the assumption that 100% of those PiC who were tobacco smokers prior to the introduction of smoke-free policy will resume tobacco smoking on release from prison, compared to 42% in the base-case

analysis. This Scenario is also impacted by the change in published utilities. The combination of these changes in parameters results in lower incremental costs and QALYs than the base-case analysis, -£900 compared to -£21,127 and 0.126 compared to 0.639. Scenario 1 *(PiC resumption of smoking on release)* which replaces 42% of PiC resuming smoking with 95%, results in smaller incremental costs due to increased costs after release for personal spend on tobacco, and smaller incremental QALYs due to increased harm from increased tobacco smoking.

The sensitivity analysis with the biggest impact on the staff ICER was Scenario 7. This is driven by higher numbers of never smokers in the base-case analysis compared to the sensitivity analysis (91% compared to around 80%), resulting in lower incremental QALYs in the sensitivity analysis (0.094 compared to 0.229), and a larger difference in utility values between pre-policy and post-policy in the base-case analysis.

Supplementary Table 12 Scenario (lifetime) analysis: sensitivity analyses results

Scenario		Presence of smoke-free policy	Absence of smoke-free policy	Incremental result
	Base-case results			
	People in custody			
Total cost		£22,399	£50,838	-£28,440
QALYs		21.781	20.810	0.971
Cost per QALY				-£29,288
	Operational staff			
Total cost		£12,343	£12,803	-£460
QALYs		29.815	29.554	0.262
Cost per QALY				-£1,760
	1) PiC smoking on release			
	People in custody			
Total cost		£44,889	£50,838	-£5,949
QALYs		20.968	20.810	0.159
Cost per QALY				-£37,512
	2) PiC time in prison: 1 year			
	People in custody			
Total cost		£24,969	£54,956	-£29,987
QALYs		21.609	20.722	0.887
Cost per QALY				-£33,824
	3) PiC time in prison: 5 years			
	People in custody			
Total cost		£20,062	£47,001	-£26,939
QALYs		21.945	20.892	1.053
Cost per QALY				-£25,581
	4) 'In prison' tobacco and e-cigarette spend: TIPs survey			

	People in custody			
Total cost		£22,919	£51,429	-£28,510
QALYs		21.781	20.810	0.971
Cost per QALY				-£29,360
	5) 'Post-prison' tobacco spend: canteen 'in prison' data			
	People in custody			
Total cost		£8,758	£12,864	-£4,107
QALYs		21.781	20.810	0.971
Cost per QALY				-£4,229
	6) Published utilities (PiC and staff)			
	People in custody			
Total cost		£22,399	£50,838	-£28,440
QALYs		23.397	22.325	1.072
Cost per QALY				-£26,528
	Operational staff			
Total cost		£12,343	£12,803	-£460
QALYs		29.850	29.729	0.121
Cost per QALY				-£3,804
7) Replacing TIPs data with published data as far as possible				
People in custody				
Total cost		£45,946	£47,107	-£1,160
QALYs		22.776	22.635	0.141
Cost per QALY				-£8,224
Operational staff				
Total cost		£17,369	£18,322	-£953
QALYs		29.460	29.352	0.109
Cost per QALY				-£8,776

PiC; Person in custody, QALY; quality adjusted life-year

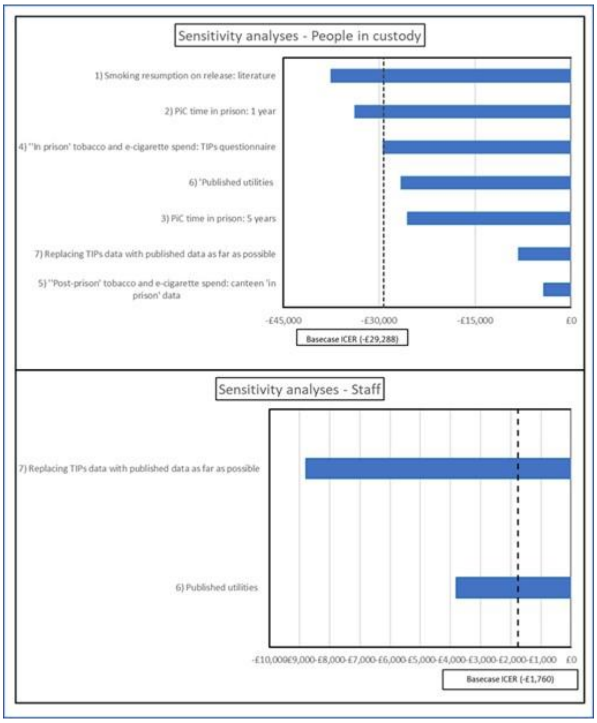

Supplementary Figure 2 Scenario (lifetime) analysis: sensitivity analyses (tornado diagram)

Table 13 Scenario (lifetime) analysis: population level analysis

	**Presence of smoke-free policy**	**Absence of smoke-free policy**	**Difference (95% confidence interval)**	**95% confidence interval**
**People in custody (n=7,500)**				
Total costs	£168,037,000	£381,288,000	-£213,251,000	-£220,113,000 to -£205,641,000
Total QALYs	163,356	156,074	7,282	3,950 to 10,208
**Staff (n=3,244)**				
Total costs	£40,041,000	£41,534,000	-£1,493,000	-£1,745,000 to -£1,210,000
Total QALYs	96,721	95,873	848	-63 to 1,844

QALYs – quality adjusted life-years

References

1. Semple S, Sweeting H, Demou E, Logan G, O'Donnell R, Hunt K, et al. Characterising the Exposure of Prison Staff to Second-Hand Tobacco Smoke. Annals of Work Exposures and Health. 2017;61(7):809-21.
2. Demou E, Dobson R, Sweeting H, Brown A, Sidwell S, O'Donnell R, et al. From Smoking-Permitted to Smokefree Prisons: A 3-Year Evaluation of the Changes in Occupational Exposure to Second-Hand Smoke Across a National Prison System. Annals of Work Exposures and Health. 2020;64(9):959-69.
3. Jones M, Smith M, Lewis S, Parrott S, Coleman T. A dynamic, modifiable model for estimating cost-effectiveness of smoking cessation interventions in pregnancy: application to an RCT of self-help delivered by text message. Addiction. 2019;114(2):353-65.
4. Carnie J, Broderick R, Cameron J, Downie D, Williams G. Scottish Prison Service 16th Prisoner Survey 2017 2017 [Available from: https://www.sps.gov.uk/Corporate/Publications/Publication-6101.aspx.
5. Sweeting H, Semple S, Demou E, Brown A, Hunt K. Predictors of opinions on prison smoking bans: Analyses of survey data from Scottish staff and prisoners. Tobacco Induced Diseases. 2019;17.
6. Filby A, Taylor M. National Institute for Health and Care Excellence - Smoking Cessation Interventions and Services 2018 [Available from: https://www.nice.org.uk/guidance/ng92/evidence/economic-modelling-report-pdf-4790596573.
7. National Center for Chronic Disease Prevention and Health Promotion (US) Office on Smoking and Health. The Health Consequences of Smoking - 50 Years of Progress: A Report of the Surgeon General. 2014.
8. Öberg M, Jaakkola M, Prüss-Üstün A, Schweizer C, Woodward A. Second-hand smoke: Assessing the burden of disease at national and local levels Geneva: World Health Organization; 2010 [Available from: https://www.who.int/quantifying_ehimpacts/publications/ebd18/en/.
9. National Records of Scotland. Vital Events Reference Tables 2018: National Records of Scotland; 2018 [Available from: https://www.nrscotland.gov.uk/statistics-and-data/statistics/statistics-by-theme/vital-events/general-publications/vital-events-reference-tables/2018/section-5-deaths.
10. Doll R, Peto R, Wheatley K, Gray R, Sutherland I. MORTALITY IN RELATION TO SMOKING - 40 YEARS OBSERVATIONS ON MALE BRITISH DOCTORS. British Medical Journal. 1994;309(6959):901-11.
11. Bardsley D, Dean L, Dougall I, Feng Q, Gray L, Karikoski M, et al. Scottish Health Survey 2017, Scottish Government 2017 [Available from: https://www.gov.scot/publications/scottish-health-survey-2017-volume-1-main-report/pages/62/.
12. Graham L, Fischbacher CM, Stockton D, Fraser A, Fleming M, Greig K. Understanding extreme mortality among prisoners: a national cohort study in Scotland using data linkage. European Journal of Public Health. 2015;25(5):879-85.
13. Spaulding AC, Eldridge GD, Chico CE, Morisseau N, Drobeniuc A, Fils-Aime R, et al. Smoking in Correctional Settings Worldwide: Prevalence, Bans, and Interventions. Epidemiologic Reviews. 2018;40(1):82-95.
14. Zlodre J, Fazel S. All-cause and external mortality in released prisoners: systematic review and meta-analysis. American journal of public health. 2012;102(12):e67-75.
15. Oberg M, Jaakkola MS, Woodward A, Peruga A, Pruess-Ustuen A. Worldwide burden of disease from exposure to second-hand smoke: a retrospective analysis of data from 192 countries. Lancet. 2011;377(9760):139-46.
16. Curtis L. Unit Costs of Health and Social Care 2018 | PSSRU 2019 [Available from: https://www.pssru.ac.uk/project-pages/unit-costs/unit-costs-2018/.
17. Foote C. Vaping kits for prisoners to cost £150,000 [Available from: https://www.bbc.co.uk/news/uk-scotland-46653366.

18. Lopez-Nicolas A, Trapero-Bertran M, Munoz C. Smoking, health-related quality of life and economic evaluation. European Journal of Health Economics. 2018;19(5):747-56.
19. Scottish Government. Criminal Proceedings in Scotland 2017-2018 2019 [Available from: https://www.gov.scot/publications/criminal-proceedings-scotland-2017-18/.
20. Full Fact. How long do murderers serve in prison? : @FullFact; 2020 [Available from: https://fullfact.org/crime/how-long-do-murderers-serve-prison/.
21. Briggs A, Claxton K, Sculpher M. Decision Modelling for Health Economic Evaluation. 2011 ed. Gray A, Briggs A, editors. New York: Oxford University Press; 2006.
22. Hatswell AJ, Bullement A, Briggs A, Paulden M, Stevenson MD. Probabilistic Sensitivity Analysis in Cost-Effectiveness Models: Determining Model Convergence in Cohort Models. Pharmacoeconomics. 2018;36(12):1421-6.
23. Scottish Prison Service. Scottish Prison Service Annual Report and Accounts 2017-2018 Posted: 23/07/2018 [Available from: https://www.sps.gov.uk/Corporate/Publications/Publication-6017.aspx.
24. Foote C. BBC Scotland - Vaping kits for prisoners to cost £150,000.
25. Öberg M, Jaakkola M, Prüss-Üstün A, Schweizer C, Woodward AJAfhwwiqepee. Second-hand smoke: Assessing the burden of disease at national and local levels. https://www.who.int/quantifying_ehimpacts/publications/ebd18/en/. Geneva: World Health Organization; 2010.
26. National Records of Scotland. Vital Events Reference Tables 2018. https://www.nrscotland.gov.uk/statistics-and-data/statistics/statistics-by-theme/vital-events/general-publications/vital-events-reference-tables/2018/section-5-deaths. 2018.
27. Doll R, Peto R, Wheatley K, Gray R, Sutherland I. Mortality in relation to smoking: 40 years' observations on male British doctors. British Medical Journal. 1994;309(6959):901-11.
28. Bardsley D, Dean L, Dougall I, Feng Q, Gray L, Karikoski M, et al. Scottish Health Survey 2017. Volume 1 Main Report. Edinburgh: Scottish Government; 2017.
29. Office of National Statistics. RPI: Ave price - Cigarettes 20 king size filter Available from: https://www.ons.gov.uk/economy/inflationandpriceindices/timeseries/czmp. Office for National Statistics; 2020.
30. Puljevic C, Segan CJ. Systematic Review of Factors Influencing Smoking Following Release From Smoke-Free Prisons. Nicotine & Tobacco Research. 2019;21(8):1011-20.
