# Supplementary material for: Implementation of a national smoke-free prison policy: an economic evaluation within the Tobacco in Prisons (TIPs) study
Source: Tob Control. 2022 Mar 7;32(6):701–8. doi: 10.1136/tobaccocontrol-2021-056991 (PMC7615232; doi:10.1136/tobaccocontrol-2021-056991)
Supplement: Supplementary data [file tobaccocontrol-2021-056991supp002.pdf]

Supplementary Table 1 Cost-consequence analysis results: costs and outcomes (base-case)

	Mean cost/outcome per person			Graphs from interrupted time series analysis
	**Pre-announcement phase**	**Preparatory phase**	**Post-implementation phase**	
**Mean cost per person per month**				
**PiC outpatient visits ł**	£3.61	£2.76	£2.48	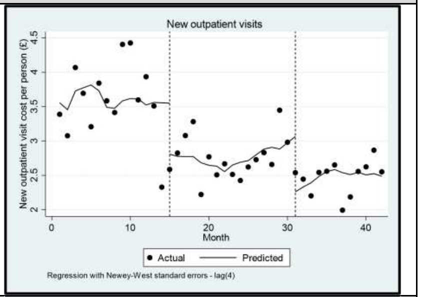
**PiC inpatient stays ł**	£10.40	£6.94	£7.71	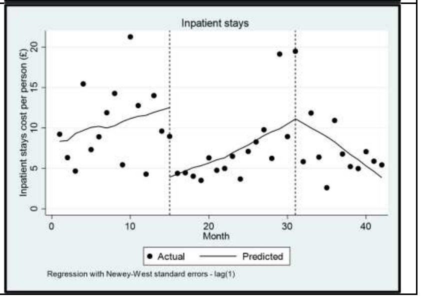

**PiC mental health hospital stays ł**	£321	£370	£217	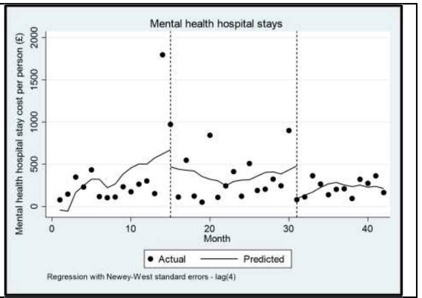
**PiC accident & emergency visits ł**	£1.53	£1.71	£1.46	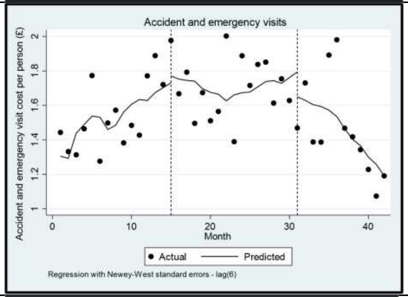
**PiC ambulance use ł**	£0.74	£1.15	£1.35	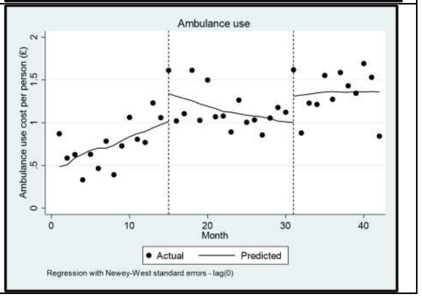

**PiC medication (nicotine dependence) ł**	£1.83	£2.39	£2.83	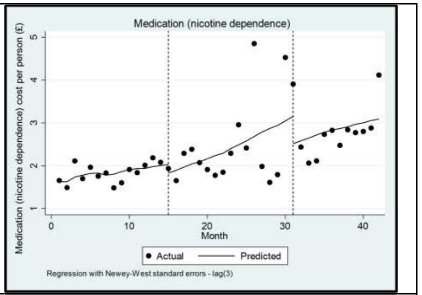
**PiC medication (smoking related illness) ł**	£2.55	£0.88	£0.79	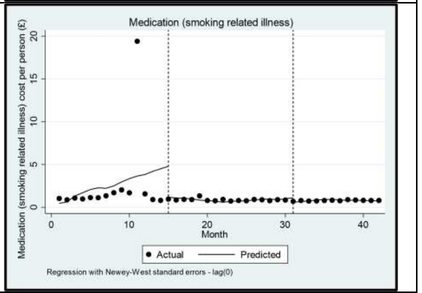
**PiC e-cigarettes ł**	N/A (not available to purchase from canteen)	£1.77	£18.26	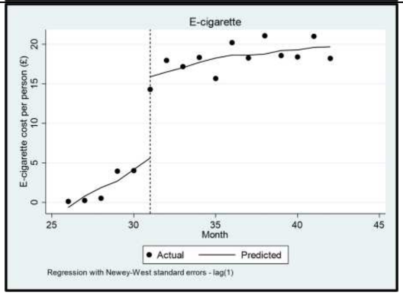
**PiC GP/nurse visits ***	£3.64 (SD 10.84) n=2354	£4.32 (SD 23.54) n=1783	£4.77 (SD 26.65) n=1336	

**Staff GP visits ***	£3.03 (SD 8.26) n=991	£2.67 (SD 7.88) n=1001	£1.99 (SD 5.89) n=513	
**PiC tobacco ***	£24.93	£24.93	N/A (no longer available to purchase from canteen)	
**Staff tobacco ***	£16.91 (SD 64.28) n=993	£16.96 (SD 58.54) n=1008	£15.98 (SD 58.08) n=514	
**Outcome per person**				
**Levels of second-hand smoke (µg/m^3) Mean PM$_{2.5}$ (SD) across all prisons (not per person)***	38.44 (SD 55.54)	11.15 (SD 4.93)	3.14 (SD 5.06)	
**Health utilities (PiC) Mean (SD) ***	0.736 (0.248) n=2268	0.725 (0.246) n=1729	0.682 (0.270) n=1298	
**Health utilities (staff) Mean (SD) ***	0.859 (0.121) n=1002	0.862 (0.127) n=925	0.863 (0.135) n=455	
**Prisoner-on-staff assaults ł †**	Data not available	0.004	0.004	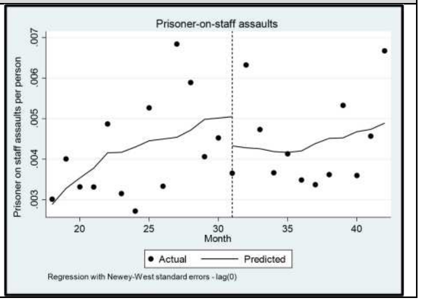

**Prisoner-on-prisoner assaults ł †**	Data not available	0.029	0.040	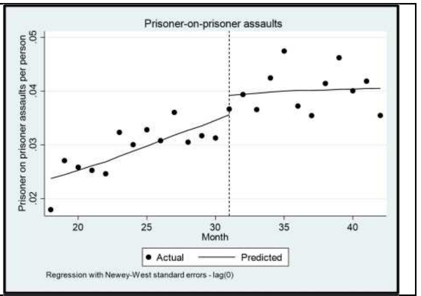
**All-cause mortality (deaths in custody) ł †**	0.0003	0.0003	0.0004	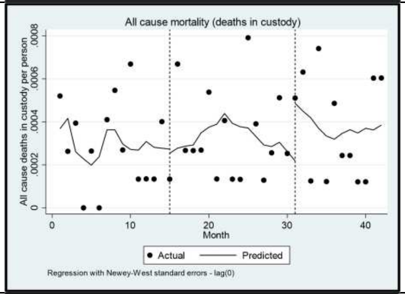
**Fires ł †**	0.0009	0.0016	0.0007	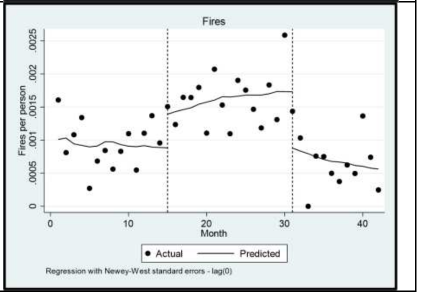

**MORS ł †**	0.011	0.036	0.046	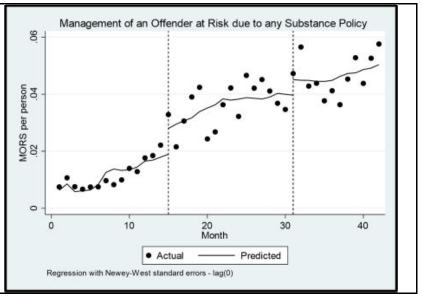

ł time series analysis; * regression framework; † mean number of events per person in custody per month; MORS - The Management of an Offender at Risk due to any Substance; PiC – person in custody; SD – standard deviation
